# Copy number variation of genes involved in the hepatitis C virus-human interactome

**DOI:** 10.1038/srep31340

**Published:** 2016-08-11

**Authors:** Lucyna Budzko, Malgorzata Marcinkowska-Swojak, Paulina Jackowiak, Piotr Kozlowski, Marek Figlerowicz

**Affiliations:** 1 Institute of Bioorganic Chemistry, Polish Academy of Sciences, Poznan, Poland; 2 Institute of Chemical Technology and Engineering, Poznan University of Technology, Poznan, Poland; 3 Institute of Computing Science, Poznan University of Technology, Poznan, Poland

## Abstract

Copy number variation (CNV) is a newly discovered form of intra-species genetic polymorphism that is defined as deletions or duplications of genome segments ranging from 1 kbp to several Mbp. CNV accounts for the majority of the genetic variation observed in humans (CNV regions cover more than 10% of the human genome); therefore, it may significantly influence both the phenotype and susceptibility to various diseases. Unfortunately, the impact of CNV on a number of diseases, including hepatitis C virus (HCV) infection, remains largely unexplored. Here, we analyzed 421 human genes encoding proteins that have been shown to interact with HCV proteins or genomic RNA (proteins from the HCV-human interactome). We found that 19 of the 421 candidate genes are located in putative CNV regions. For all of these genes, copy numbers were determined for European, Asiatic and African populations using the multiplex ligation-dependent amplification (MLPA) method. As a result, we identified 4 genes, *IGLL1*, *MLLT4*, *PDPK1*, *PPP1R13L,* for which the CN-genotype ranged from 1 to 6. All of these genes are involved in host-virus interaction; thus, their polymorphism has a potential impact on the development of HCV infection and/or therapy outcome.

The hepatitis C virus (HCV), discovered in 1989, is a hepatotropic, positive-sense, single-stranded (ss) RNA virus that belongs to the family *Flaviviridae*^1^. The HCV genome encodes 10 mature viral proteins, including 3 structural proteins (C, E1 and E2) and 7 nonstructural proteins (p7, NS2, NS3, NS4A, NS4B, NS5A, and NS5B)^2^. The potential consequences of HCV infection vary in severity from a transient and symptomless illness to a mortal, lifelong disease. Approximately 15–45% of infected persons spontaneously clear the virus without treatment during the acute phase of the infection. The remaining individuals develop chronic hepatitis C (CHC). Globally, an estimated 130–150 million people are chronically infected with HCV. Approximately 15–30% of patients with CHC develop cirrhosis over a period of 20 years. Those with cirrhosis have a poor prognosis due to a high risk of liver failure and hepatocellular carcinoma (HCC). Consequently, nearly 500 000 people die each year from hepatitis C-related liver diseases^3^.

Since the discovery of HCV, a number of studies have aimed to identify human- and virus-specific factors that affect the course of HCV infection and patient response to antiviral treatment. The majority of these studies have focused on the three general issues: (i) the genetic variability of HCV, (ii) the clinical status of the patient, and (iii) the genetic variation of the human population.

HCV, similar to other RNA viruses, displays an extremely high level of genetic variability. HCV is classified into 7 genotypes (numbered 1–7), which are further divided into more than 60 subtypes^4^,^5^. Genotype 1 is the most common worldwide (46.2% of infected individuals), followed by genotype 3 (30.1%) and genotypes 2, 4 and 6 (22.8% collectively). Genotypes 5 and 7 comprise the remaining 0.9%^6^. The major source of HCV genetic variability is the highly efficient (10^12^ virions per day) but low-fidelity replication^5^ by viral RNA-dependent RNA polymerase (NS5B) that lacks proofreading activity (this enzyme exhibits an error rate of 8.7 × 10^−3^−1.4 × 10^−6^ per site)^2^,^7^,^8^. The consequence of this continuous variation in the HCV genome is a presence in a single host of a collection of closely related, but genetically divergent, viral variants called a quasispecies^9^,^10^. It has been shown that susceptibility to antiviral treatment correlates with HCV variability on both the genotype and quasispecies level. Furthermore, there are a number of reports that show a genotype-specific response in patients treated with interferon and ribavirin (IFN-RBV)^5^,^11^ a standard HCV treatment before 2013^12^. The effect of genotype, however, seems to be less prominent in the case of currently used therapies that are based on direct-acting antiviral agents (DAAs)^13^. In addition, analyses of viral populations isolated from individual patients have revealed a correlation between the complexity of HCV quasispecies and the outcome of CHC therapy^14^,^15^,^16^,^17^,^18^. Other studies have indicated that the course of viral infection and the patient’s response to the antiviral treatment may be influenced by specific mutations within the viral genome^19^,^20^. Moreover, previous data from our laboratory indicated the existence of HCV variants with higher fitness, which are capable of persisting in epidemiologically unrelated hosts^14^,^21^.

The clinical status of the patient is the second important determinant of the course of HCV infection and therapeutic outcome. The factors that have the most significant association with poor prognosis in HCV infection are HCV/HIV co-infection, alcoholism, immune system disorders as well as HCV-related and non-related oncogenesis^22^,^23^,^24^,^25^,^26^.

Human genetic variation is also considered one of the most significant factors that underlie the unique susceptibility of each person to HCV infection and antiviral treatment. Genome-wide association studies (GWAS) have identified that single nucleotide polymorphisms (SNPs) near the *IL28B* gene are strongly associated with both the spontaneous and treatment-induced clearance of HCV infection^27^,^28^,^29^. SNPs of this gene are also one of the factors underlying the observed differences in sustained virological response (SVR) rate to IFN-RBV treatment in various human populations^27^. Recently, another type of intra-species polymorphism with significant biological implications, called copy number variation (CNV), has been discovered. CNV is caused by DNA alterations that result in copy number (CN) changes of a particular DNA segment larger than 1 kb. The location of the protein-coding gene within CNV region (which is frequently observed in humans) may affect gene dosage and/or alternate gene structure. Consequently, CNV may significantly influence the phenotypes^30^,^31^,^32^,^33^ and contribute to individual variation in drug response, immune defense, disease resistance or susceptibility. The most prominent example of a CNV-based gene dosage variation with clinical implication are CN changes of *CCL3L1* (chemokine (C-C motif) ligand 3-like 1) – a gene encoding chemokine that is a ligand to the co-receptor for human immunodeficiency virus (HIV). It has been found that the CN of this gene ranges from 0 to 14 in diploid human genomes^34^,^35^. In addition, it has been reported that the lower CN of *CCL3L1* is an important genetic determinant for increased HIV-1 susceptibility and faster AIDS progression^36^. However, there are many controversies and conflicting results regarding this association^37^,^38^,^39^,^40^ (discussed in ref. 41). It was suggested that observed discordances result mostly from the lack of appropriate CNV genotyping method allowing unequivocal separation of CN-genotypes. It has also been shown that lower CN of the *CCL3L1* gene, compared to the population median, is associated with susceptibility to CHC^32^,^42^. According to our best knowledge, this is the only known example of an association between a course of HCV infection and CN changes of a human gene.

In this study, we identified genes that encode proteins belonging to the HCV-human interactome that undergo CN changes and whose dosage effect could potentially impact the course of HCV infection and/or treatment outcome. After gene identification, we experimentally characterized 19 of them in individuals from different human populations and proved the polymorphic nature of four of them. Our data also confirmed previous reports regarding *CCL3L1* CN distribution among different populations^30^,^32^,^35^.

## Results

### Selection of genes

**Figure 1:**
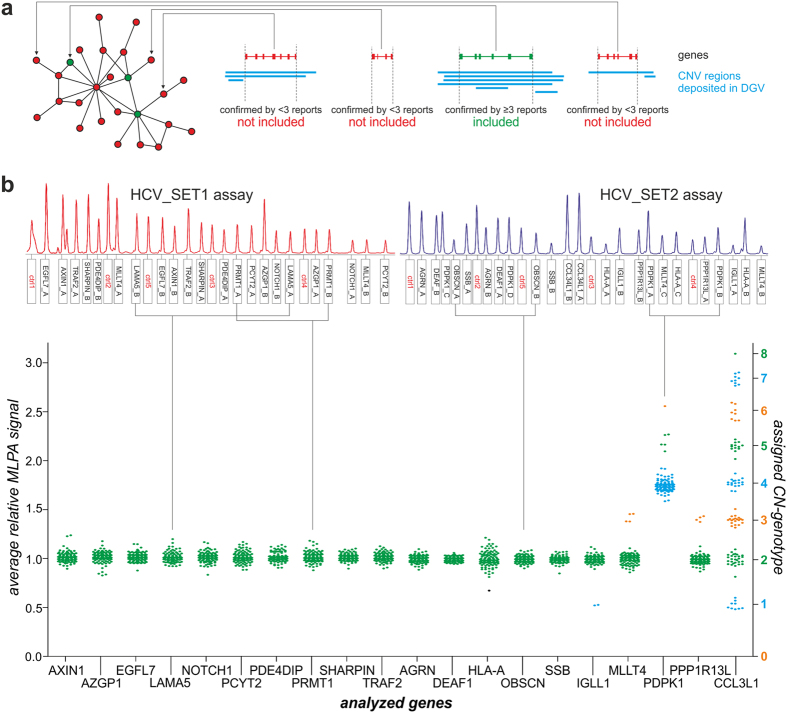
MLPA analysis of selected genes. (**a**) Gene selection strategy. Schematic representation (on the left) shows examples of human genes involved in the HCV-human interactome network^43^. The gene was included in our analysis if the overlap between this gene (green) and CNV region deposited in DGV (blue) was confirmed by at least 3 independent reports. Other genes (red) were not selected for analysis in this study. (**b**) MLPA analysis. Presented electropherograms (upper panel) show two MLPA assays designed for analysis of selected genes: HCV_SET1 assay (red) and HCV_SET2 assay (blue). Probe IDs are indicated under the electropherograms. Control probes are indicated in red. One-dimensional signal scatter-plot (bottom panel) shows an average relative signal of gene specific MLPA probes (left *y*-axis) and assigned CN-genotypes (right *y*-axis) for selected genes (IDs indicated on *x*-axis) in all analyzed samples. One dot represents one sample, colored in accordance with assigned CN-genotype (right y-axis). A black dot represents a sample with no CN-genotype assigned.

To identify candidate human genes overlapping with CNVs for which the dosage effect could correlate with a course of HCV infection and/or treatment outcome, we focused on 421 genes (Supplementary Table S1) encoding proteins that have been shown to interact with HCV proteins or HCV genome^43^ (the most comprehensive dataset of HCV-human interactome available when we began our studies). To determine whether these genes co-localize with known CNV regions, we searched the Database of Genomic Variants (DGV)^44^, which collects the data regarding structural variation that have been reported to date. For our analysis, we selected only the genes for which the overlaps with CNV regions were confirmed in at least 3 independent publications (Fig. 1a). As a result, we identified 19 candidate genes (Supplementary Table S2) and selected them for further experimental studies. Additionally, we added *CCL3L1*, a highly polymorphic gene, to the group of the selected genes to serve as a positive control.

### CNV analysis

To analyze the 20 selected genes, we applied the multiplex ligation-dependent amplification (MLPA)^45^ method and the strategy of probes and assays design that we successfully used before^46^,^47^. For each gene, we designed two MLPA probes. For genes localized in more complex genomic regions, we designed additional probes: (i) one for *HLA-A* (major histocompatibility complex, class I, A) and *MLLT4* (myeloid/lymphoid or mixed-lineage leukemia; translocated to, 4) and (ii) two for *PDPK1* (3-phosphoinositide dependent protein kinase 1). All probes were split between two MLPA assays named: HCV_SET1 and HCV_SET2. The HCV_SET1 assay consisted of 22 probes that were specific for 11 of the selected genes, and the HCV_SET2 assay included 23 probes that were specific for 10 of the selected genes. Each assay also contained five control probes, specific to CN stable regions, which were used for the normalization of the run-to-run MLPA probe signal variation. The detailed features of all MLPA probes and assays layout are shown in Supplementary Table S3.

Using both assays, we analyzed the CN changes of the selected genes in 106 DNA samples from three different human populations: 31 European (CEU) samples, 48 Asiatic (CHB/JPT) samples and 27 African (YRI) samples.

The representative results of MLPA analysis (MLPA signal electropherograms) are presented in Fig. 1b (upper panel). The results obtained for all of the analyzed genes in all samples are summarized in signal scatter plot (Fig. 1b, bottom panel). The data presented in Fig. 1b indicated that the following four of the 19 total analyzed human genes showed variation in CN: (i) *IGLL1* (immunoglobulin lambda like polypeptide 1), (ii) *MLLT4*, (iii) *PDPK1*, and (iv) *PPP1R13L* (protein phosphatase 1 regulatory subunit 13 like); the observed CN-genotypes (distinct signal clusters) ranged from 1 to 6 copies. The selected positive control gene, *CCL3L1*, also proved to be polymorphic, with CN range from 1 to 8, which was consistent with our previous results^35^. Other genes proved to be non-polymorphic and showed only one cluster corresponding to 2 CN-genotype. The list of the assigned CN-genotypes for all of the samples is shown in Supplementary Table S4.

### The extent of CNV of polymorphic genes

For all polymorphic genes we observed the occurrence of several (≥2) CN-genotypes, one of which was predominant (major CN-genotype); the alternative genotypes were represented only by a small number of samples (minor CN-genotypes). For three polymorphic genes, we observed duplications (*PPP1R13L* and *MLLT4* genes) or deletions (*IGLL1* gene) of one of the gene copies, resulting in a higher (3) and lower (1) CN compared to major CN-genotype, respectively. For these genes, the European population was the most variable, with 7 (22.6%) samples presenting the minor CN-genotype. For the *PDPK1* gene, we observed an increased CN for the major genotype (CN=4) with additional duplications that resulted in minor CN-genotypes of 5 and 6 copies. There was no sample demonstrating the minor CN-genotype for more than one gene.

**Figure 2:**
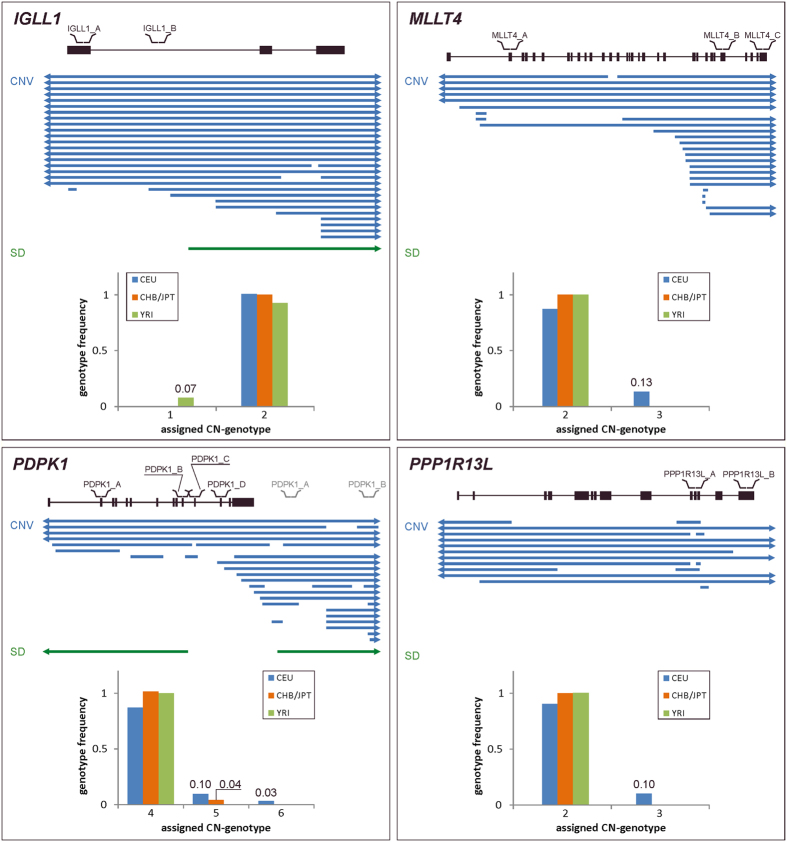
Detailed characteristics of all polymorphic genes identified in this study. Each panel shows the map of the gene with localization of all probes indicated over the map. Below the map, common structural variants available in DGV database are shown: CNV (blue) and SD (green). Arrowheads indicate that CNV/SD exceed the area shown in the figure. Bar graphs (bottom) show the frequency (*y*-axis) of a particular CN-genotype (*x*-axis) in analyzed samples from different human populations: CEU (blue), CHB/JPT (orange) and YRI (green). Note that due to the *PDPK1* gene overlap with SD, two probes (PDPK1_A and PDPK1_B) map to two genomic locations, within and downstream of *PDPK1*. The probes mapping out of the gene sites are shown in grey to distinguish them from those mapping within the gene.

According to the DGV, all of the designed MLPA probes were located within the CNV regions overlapping selected genes. However, a detailed analysis of these regions revealed that two of the regions have more complex structures (Fig. 2). The results obtained for the three probes designed for the *MLLT4* gene showed that two of the probes (MLLT4_B and MLLT4_C) targeted the polymorphic region and that the third one (MLLT4_A) targeted a non-polymorphic fragment of the gene. This result suggested that not the entire gene is located within the polymorphic region but only several exons at its 3′ end and only that part of the gene underwent CN changes in the studied samples. The structure of the region where the *PDPK1* gene is localized is even more complex. Two of the probes designed for this gene (PDPK1_C and PDPK1_D) showed no polymorphism, whereas the other two probes (PDPK1_A and PDPK1_B) showed a variable number of copies. This result suggested that the polymorphic region covers only several exons at the 5′ end and that the exons at the 3′ end appear to be located outside this region. Additionally, probes PDPK1_A and PDPK1_B co-localized with segmental duplication (SD), therefore, during the MLPA experiments they hybridized to two sites, within and downstream of *PDPK1* gene, thus producing a higher MLPA signal and, consequently, higher CN-genotypes.

## Discussion

In this study, we used bioinformatics methods to identify all human genes that both (i) encode proteins that belong to the HCV-human interactome^43^ and (ii) according to at least 3 independent reports co-localize with known CNV regions (listed in the DGV^44^). It is expected that the structural polymorphism of these genes may impact the course of HCV infection and/or treatment outcome. The above criterion was fulfilled by 19 genes, for which the changes in CN were determined using the MLPA in a cohort of 106 individuals from 3 different populations. As a result, we identified 4 polymorphic genes (*IGLL1*, *MLLT4*, *PDPK1*, *PPP1R13L*) that showed variations in CN ranging from 1 to 6 copies among the tested samples. The detailed characteristics of the structure of the polymorphic genes revealed that two of them (*MLLT4* and *PDPK1*) were partially overlapped by CNV regions. For *MLLT4*, our analysis suggested that only terminal exons at the 3′ end were located within the CNV region. In the case of *PDPK1,* the CNV region covered ten initial gene exons at the 5′ end but not the 3′ terminal ones. *IGLL1* and *PPP1R13L* were overlapped by CNV regions, at least in the parts where the probes were located. Three of the four identified polymorphic genes (*IGLL1*, *PPP1R13L*, *MLLT4)* showed CN=2 for the major CN-genotype (predominant genotype among populations). The polymorphic region of the *PDPK1* gene co-localized with segmental duplication, thus giving a higher MLPA signal equal to CN=4 for the major CN-genotype. The genetic variability identified for *IGLL1* was a deletion of one gene copy in samples from the African population. For the *MLLT4* and *PPP1R13L* genes, we observed duplication - one additional copy in samples from the European population. For *PDPK1,* additional one or two copies resulted in 5 or 6 CN-genotype for samples from the Asiatic and European population.

The functions of the identified polymorphic genes suggest that they may have an impact on the individual susceptibility to develop HCV infection. In the case of genes that are completely overlapped by polymorphic regions, changes in CN may influence the phenotype by affecting the gene dosage, whereas a partial overlap between the gene and CNV region likely does not directly influence the full transcript dosage. The latter, however, may cause changes in the transcription regulation (altered number of regulatory elements) or induce the formation of alternative transcript variants. Moreover, each CNV may affect the phenotype, regardless of gene dosage, through a position effect or a change in the genome structure.

*IGLL1* encodes immunoglobulin lambda-like polypeptide 1 protein, which is a critical B cell development receptor found on the surface of pro-B and pre-B cells. It has been shown that IGLL1 interacts with the HCV NS5A protein^43^. Dysfunctions of the *IGLL1* gene result in a primary immunodeficiency caused by poorer proliferation and differentiation of pro-B cells and consequently, lower levels of serum antibodies and circulating B cells^48^,^49^,^50^. Therefore, the observed deletion of one copy of the gene in the African population suggests that the dosage effect may potentially result in poorer immunological response.

The *PPP1R13L* gene encodes a protein phosphatase 1 regulatory subunit 13-like protein, called iASPP protein, which inhibits apoptosis and regulates transcription by interacting with NF-kappa-B and p53/TP53 proteins^51^,^52^. It has also been shown that *PPP1R13L* blocks the transcription of HIV-1 by inhibiting the action of both NF-kappa-B and constitutive transcription factor SP1^53^. Interestingly, HCV represses apoptosis^54^, and the activation of NF-kappa-B^55^ and SP1^56^,^57^ in CHC is significantly modulated by viral proteins. Moreover, the direct interaction of iASPP protein and HCV NS5A (a phosphoprotein that plays a key role in HCV RNA replication^58^) has been shown^43^. The effects of viral proteins on apoptosis pathways, NF-kappa-B and SP1 functions in relation to the course of HCV infection are complex. Nevertheless, in this context, the additional copy of the *PPP1R13L* gene observed in samples from the European population may be significant for the progression of liver injury and the severity of CHC.

Among the four identified polymorphic genes, the initial exons at 5′ end of *PDPK1* showed the highest variability in the tested populations, ranging from 4 to 6 copies. *PDPK1* encodes 3-phosphoinositide-dependent protein kinase 1 (hPDK1), a serine/threonine kinase that acts as a master kinase that phosphorylates and activates a subgroup of the AGC family of protein kinases (PKA, PKC, PKG). Thus, hPDK1 plays a central role in the transduction of signals to downstream targets controlling cell proliferation and survival. For example, it negatively regulates the TGF-beta-induced signaling and activates the NF-kappa-B pathway^59^,^60^. Its interaction with the HCV NS5A protein has been shown^43^. Moreover, hPDK1 is an upstream kinase of the protein kinase C-related kinase 2 (PRK2) that is responsible for the phosphorylation of HCV RNA polymerase. Destabilization of hPDK1 suppresses hepatitis C virus replication^61^. Our results demonstrated that the CNV region overlaps the initial exons of the *PDPK1* gene (and potentially the promoter region). Duplications of this fragment of the *PDPK1* gene may result in the expression of alternative (shorter) transcripts or have other indirect functional impacts. The high variability of the *PDPK1* gene-overlapping CNV region among populations and functional relevance of hPDK1 for HCV replication strongly encourages more detailed structural and functional studies.

The last identified polymorphic gene, *MLLT4*, encodes Afadin (AF6) a multi-domain protein involved in signaling and organization of cell junctions. Together with the E-cadherin-catenin system, Afadin belongs to the adhesion system that plays a role in the organization of homotypic, interneuronal and heterotypic cell-cell adherens junctions^62^. It has been shown that Afadin interacts with HCV nonstructural NS3 protein^43^. The observed additional copy of the terminal exons of the gene in samples from the European population would not directly affect the gene dosage but may have an indirect phenotypic consequence via a position effect or the altered regulation of the gene expression.

CN of the *CCL3L1* gene has been previously shown to differ significantly between populations^32^,^34^,^35^,^36^. Therefore, we included *CCL3L1* to the group of studied genes and used it as a positive control. Our experiments confirmed extensive CNV of *CCL3L1* and specific distribution of CN-genotypes among different population. We noted a *CCL3L1* CN-genotype range of 1–3 copies in the European population, 2–8 copies in the Asiatic population and 2–7 copies in the African population.

The impact of CNV on phenotype and its association with diseases remain poorly understood. To date, transcriptome analyses and single-gene expression studies are appropriate proxies to study the physiological and pathological consequences of CNV. Here, we present a selection of candidate human genes overlapped by CNV regions for further functional investigations as potential genetic markers. CNVs of these four newly identified polymorphic genes may serve in the future as potential predictors of CHC development, spontaneous virus clearance or response to treatment. These results also enhance our understanding of host genetic factors that influence pathogenesis of HCV infection.

Summarizing, in this paper we present a simple approach that permits to identify genes whose CNV may affect host-virus interactions. The proposed approach combines the data on CNV regions present in the human genome and host-virus interactome to select candidate genes. In the next step, variation in CN of each selected gene is experimentally verified by the MLPA method. The analysis described here focused on HCV, a model human RNA virus, however, there is no doubt that the same procedure can be used to determine the influence of CNV on other viral infections.

Our analysis revealed also some weaknesses of the data sets being at our disposal. Although, we applied very restrictive selection criteria only 4 out of 19 candidate genes from HCV-host interactome showed CNV in the tested set of DNA samples. Thus, more precise characterization of both CNV regions in human genome and HCV-human interactome is still necessary to better elucidate the associations between CNV and the course of HCV infection. The problem of HCV-host interactions has been recently addressed by several research groups^63^,^64^,^65^. As a result, the size of the interactome has been increased from 421 to 969 human proteins^64^. Considering the rapid progress currently observed in the studies on human genome polymorphism one can expect that the approach presented here, will be broadly applied to identify new genetic factors shaping host-virus interactions.

## Methods

### Gene selection

To identify the human genes whose overlap with CNV regions could possibly correlate with a course of HCV infection and/or treatment outcome, we compared the genomic localization of 421 genes encoding proteins belonging to human-HCV interactome^43^ and 202 430 CNV regions from the Database of Genomic Variants (updated July 23, 2013; www.dgv.tcag.ca). As genes that are potentially affected by CNV, we selected genes that overlapped with at least three CNV regions deposited in the DGV database.

In addition to genes from the human-HCV interactome, we selected one gene (*CCL3L1*) as a positive control that fulfilled the following criteria: (i) it has been proven to be highly polymorphic among different human populations^35^, and (ii) its CNV was shown to be associated with the course of HCV infection^42^.

### Samples

In this study, 106 DNA samples from three different worldwide human populations were used: 31 European (CEU) samples from CEPH/Utah Collection, 48 Asiatic (CHB/JPT) samples from Han Chinese in Beijing (China) and Japanese in Tokyo (Japan), and 27 African (YRI) samples from Yoruba in Ibadan (Nigeria). All samples belong to the HapMap Project samples and were obtained from Coriell Institute (www.coriell.org).

### MLPA probes and assays design

For the CNV analyses of 20 selected genes, two custom-made assays (sets of MLPA probes designed for the analyzed genes) named: HCV_SET1 and HCV_SET2, were designed. The HCV_SET1 assay contained 22 probes specific for 11 selected genes, and the HCV_SET2 assay contained 23 probes specific for 10 genes. Both assays also contained five control probes designed for CN stable regions of known number of copies (CN=2). The total probe length ranged from 93 to 198 and from 93 to 192 nucleotides in the HCV_SET1 and HCV_SET2 assay, respectively. All MLPA probes were designed according to a previously described strategy^46^,^66^ to avoid known SNPs, repeat elements and sequences of extremely high or low GC content. Probes were predominantly designed to be located in exons. For each gene, at least two MLPA probes were designed. For *MLLT4* and *HLA-A* genes, one additional probe was designed, and for the *PDPK1* gene, two additional probes were designed due to the complex genomic structure of the regions where these genes are located. For *PDPK1* two probes were designed in a unique part of the gene (PDPK1_C, PDPK1_D), while the other two (PDPK1_A, PDPK1_B) were located in the part that overlaps with segmental duplication (SD) (Fig. 2). The latter two, due to the high homology of present SDs, were designed as universal, i.e. they recognize both sequences, within and downstream of the gene.

The detailed characteristics and sequences of all probes used in this study are presented in Supplementary data (Table S3). All probes were generated by Integrated DNA Technologies Inc. (IDT, Coralville, IA, USA; www.idtdna.com) in 100 nmole scale and purified by PAGE.

### MLPA analysis

MLPA reactions were performed according to published results^45^ and the manufacturer’s protocol (MRC-Holland, Amsterdam, Netherlands; www.mrc-holland.com). All of the MLPA reagents, except for probe-mixes (which were prepared separately from designed and generated oligonucleotides), were purchased from MRC-Holland.

In brief, 100 ng of sample DNA was denatured and hybridized with MLPA probe-mix for 16 hours. All probes that correctly hybridized to their targets were ligated and amplified with the use of universal primer pair. PCR products were separated by capillary electrophoresis on ABI Prism 3130XL Analyzer (Applied Biosystems, Carlsbad, CA, USA). Obtained electropherograms were analyzed with the use of GeneMarker software v1.91 (SoftGenetics, State College, PA, USA; www.softgenetics.com) and signal intensity values (peak heights) were transferred to prepared Microsoft Excel sheets. The signals of all probes were first normalized. To this end, each individual signal was divided by the average signal of control probes to avoid run-to-run signal variation. Then, the normalized MLPA signal of all probes specific for a particular gene was averaged and presented in one-dimensional (1D) signal scatter plot. Because the MLPA signal is proportional to CN of tested region/gene, the plot signals from all analyzed samples formed distinct clusters representing CN-genotypes, which were assigned according to previously described method^35^,^47^.

All graphs and statistical analyses were performed using Microsoft Excel and GraphPad Prism v.5.00.

## Additional Information

**How to cite this article**: Budzko, L. *et al*. Copy number variation of genes involved in the hepatitis C virus-human interactome. *Sci. Rep.* **6**, 31340; doi: 10.1038/srep31340 (2016).

## Supplementary Material

Supplementary Figures

Supplementary Table 1

Supplementary Table 2

Supplementary Table 3
