# Supplementary material for: Copy number variation of genes involved in the hepatitis C virus-human interactome
Source: Sci Rep. 2016 Aug 11;6:31340. doi: 10.1038/srep31340 (PMC4980658; doi:10.1038/srep31340)
Supplement: Supplementary Figures [file srep31340-s1.pdf]

Supplementary Information

Copy number variation of genes involved in the hepatitis C virus-human interactome

Lucyna Budzko[1,+], Malgorzata Marcinkowska-Swojak[1,+], Paulina Jackowiak[1,2],

Piotr Kozlowski[1,2], Marek Figlerowicz[1,3,]*

[1] Institute of Bioorganic Chemistry, Polish Academy of Sciences, Poznan, Poland

[2] Institute of Chemical Technology and Engineering, Poznan University of Technology, Poznan, Poland

[3] Institute of Computing Science, Poznan University of Technology, Poznan, Poland

[+] These authors contributed equally to this work.

* Corresponding author

Prof. Marek Figlerowicz

Institute of Bioorganic Chemistry

Polish Academy of Sciences

Noskowskiego 12/14

61-704 Poznan, Poland

E-mail: marekf@ibch.poznan.pl

Supplementary Table S1. List of genes encoding proteins belonging to HCV-human interactome (see .xls file).

Supplementary Table S2. Characteristic of 19 candidate genes selected for MLPA experiment.

No.	gene	chr	reported CNV	
			coverage of the entire RefSeq gene	partial coverage (>50% of the gene length)
1.	**AGRN***	chr1	[1-11]	[12]
2.	**AXIN1**	chr16	[13-15]	[8,12]
3.	**AZGP1**	chr7	[4,12,16]	[10,17]
4.	**DEAF1**	chr11	[3,15,18,19]	[1,12,13]
5.	**EGFL7**	chr9	[1,3,5,8,11,12,14,19,20]	[17]
6.	**HLA-A**	chr6	[1,3-5,9-12,16-18,20-29]	[30]
7.	**IGLL1**	chr22	[1,5,7,9,11-13,19,20,22,24,26]	[8,10,16,31]
8.	**LAMA5**	chr20	[7,14,15,19]	[12,20,32]
9.	**MLLT4**	chr6	[9,14,23]	[1,3,15]
10.	**NOTCH1**	chr9	[3,8,19,33]	[5,11,12,20,26]
11.	**OBSCN**	chr1	[4,7,17]	[1,12,19]
12.	**PCYT2**	chr17	[6,8,15-17,19,33]	—
13.	**PDE4DIP**	chr1	[1,3,6,8,18,21,26,31]	[4,9-11,15-17,24,34-37]
14.	**PDPK1**	chr16	[1,3,14,15,26]	[10,16]
15.	**PPP1R13L**	chr19	[5,12,15,20,24]	[1,13]
16.	**PRMT1**	chr19	[5,8,15,19,32,35]	—
17.	**SHARPIN**	chr8	[1,3,6-8,12,19,20,33]	—
18.	**SSB**	chr2	[1,5,12,26]	—
19.	**TRAF2**	chr9	[3,7,8,14,19,32]	—

(*) Genes are referenced with their cognate NCBI gene name; chr – chromosome

Supplementary Table S3. Characteristics of all designed MLPA probes and assays layout (see .xls file). Sheet 1 – HCV_SET1, custom-made MLPA assay for CNV analysis of 11 selected genes. Sheet 2 – HCV_SET2, custom-made MLPA assay for CNV analysis of 10 selected genes.

Supplementary Table S4. Full list of all assigned genotypes in this study (see .xls file).

Supplementary References

1. Coe, B. P. et al. Refining analyses of copy number variation identifies specific genes associated with developmental delay. *Nat Genet* **46,** 1063-71 (2014).
2. Iafrate, A. J. et al. Detection of large-scale variation in the human genome. *Nat Genet* **36,** 949-51 (2004).
3. Redon, R. et al. Global variation in copy number in the human genome. *Nature* **444,** 444-54 (2006).
4. Park, H. et al. Discovery of common Asian copy number variants using integrated high-resolution array CGH and massively parallel DNA sequencing. *Nat Genet* **42,** 400-5 (2010).
5. Perry, G. H. et al. The fine-scale and complex architecture of human copy-number variation. *Am J Hum Genet* **82,** 685-95 (2008).
6. Perry, G. H. et al. Copy number variation and evolution in humans and chimpanzees. *Genome Res* **18,** 1698-710 (2008).
7. Shaikh, T. H. et al. High-resolution mapping and analysis of copy number variations in the human genome: a data resource for clinical and research applications. *Genome Res* **19,** 1682-90 (2009).
8. Dogan, H., Can, H. & Otu, H. H. Whole genome sequence of a Turkish individual. *PLoS One* **9,** e85233, doi: 10.1371/journal.pone.0085233 (2014).
9. Vogler, C. et al. Microarray-based maps of copy-number variant regions in European and sub-Saharan populations. *PLoS One* **5,** e15246, doi:10.1371/journal.pone.0015246 (2010).

10. Sudmant, P. H. et al. Evolution and diversity of copy number variation in the great ape lineage. *Genome Res* **23,** 1373-82 (2013).
11. Teague, B. et al. High-resolution human genome structure by single-molecule analysis. *Proc Natl Acad Sci USA* **107,** 10848-53 (2010).
12. Cooper, G. M. et al. A copy number variation morbidity map of developmental delay. *Nat Genet* **43,** 838-46 (2011).
13. Mills, R. E. et al. Mapping copy number variation by population-scale genome sequencing. *Nature* **470,** 59-65 (2011).
14. Simon-Sanchez, J. et al. Genome-wide SNP assay reveals structural genomic variation, extended homozygosity and cell-line induced alterations in normal individuals. *Hum Mol Genet* **16,** 1-14 (2007).
15. Wong, K. K. et al. A comprehensive analysis of common copy-number variations in the human genome. *Am J Hum Genet* **80,** 91-104 (2007).
16. Conrad, D. F. et al. Origins and functional impact of copy number variation in the human genome. *Nature* **464,** 704-12 (2009).
17. de Smith, A. J. et al. Array CGH analysis of copy number variation identifies 1284 new genes variant in healthy white males: implications for association studies of complex diseases. *Hum Mol Genet* **16,** 2783-94 (2007).
18. Abecasis, G. R. et al. An integrated map of genetic variation from 1,092 human genomes. *Nature* **491,** 56-65 (2012).
19. Jakobsson, M. et al. Genotype, haplotype and copy-number variation in worldwide human populations. *Nature* **451,** 998-1003 (2008).
20. Itsara, A. et al. Population analysis of large copy number variants and hotspots of human genetic disease. *Am J Hum Genet* **84,** 148-61 (2009).

21. Abecasis, G. R. et al. A map of human genome variation from population-scale sequencing. *Nature* **467,** 1061-73 (2010).

22. Wong, L. P. et al. Deep whole-genome sequencing of 100 southeast Asian Malays. *Am J Hum Genet* **92,** 52-66 (2013).

23. Pinto, D., Marshall, C., Feuk, L. & Scherer, S. W. Copy-number variation in control population cohorts. *Hum Mol Genet* **16 Spec No. 2,** R168-73 (2007).

24. Uddin, M. et al. A high-resolution copy-number variation resource for clinical and population genetics. *Genet Med* **17,** 747-52 (2014).

25. McCarroll, S. A. et al. Integrated detection and population-genetic analysis of SNPs and copy number variation. *Nat Genet* **40,** 1166-74 (2008).

26. Kidd, J. M. et al. Mapping and sequencing of structural variation from eight human genomes. *Nature* **453,** 56-64 (2008).

27. Ahn, S. M. et al. The first Korean genome sequence and analysis: full genome sequencing for a socio-ethnic group. *Genome Res* **19,** 1622-9 (2009).

28. Gusev, A. et al. Whole population, genome-wide mapping of hidden relatedness. *Genome Res* **19,** 318-26 (2009).

29. Boomsma, D. I. et al. The Genome of the Netherlands: design, and project goals. *Eur J Hum Genet* **22,** 221-7 (2013).

30. Pang, A. W. et al. Towards a comprehensive structural variation map of an individual human genome. *Genome Biol* **11,** R52 (2010).

31. Korbel, J. O. et al. Paired-end mapping reveals extensive structural variation in the human genome. *Science* **318,** 420-6 (2007).

32. Wang, K. et al. PennCNV: an integrated hidden Markov model designed for high-resolution copy number variation detection in whole-genome SNP genotyping data. *Genome Res* **17,** 1665-74 (2007).

33. Levy, S. et al. The diploid genome sequence of an individual human. *PLoS Biol* **5,** e254, doi: 10.1371/journal.pbio.0050254 (2007).

34. Ju, Y. S. et al. Reference-unbiased copy number variant analysis using CGH microarrays. *Nucleic Acids Res* **38,** e190, doi: 10.1093/nar/gkq730 (2010).

35. Locke, D. P. et al. Linkage disequilibrium and heritability of copy-number polymorphisms within duplicated regions of the human genome. *Am J Hum Genet* **79,** 275-90 (2006).

36. Sebat, J. et al. Large-scale copy number polymorphism in the human genome. *Science* **305,** 525-8 (2004).

37. Sharp, A. J. et al. Segmental duplications and copy-number variation in the human genome. *Am J Hum Genet* **77,** 78-88 (2005).
